# Catalytic Performance of Highly Dispersed Bimetallic Catalysts for CO Hydrogenation to DME

**DOI:** 10.1002/cplu.202500010

**Published:** 2025-03-12

**Authors:** Chunqiu Zhao, Qiang Chang, Fu Yin, Guowei Niu, Chenghua Zhang, Dan Liu, Bhekie B. Mamba, Alex T. Kuvarega

**Affiliations:** ^1^ Institute for Nanotechnology and Water Sustainability (iNanoWS) College of Science Engineering and Technology (CSET) University of South Africa, Florida Science Campus Johannesburg 1710 South Africa; ^2^ National Energy Center for Coal to Liquids Synfuels China Co., Ltd. Huairou District Beijing 101400 China; ^3^ Tianjin Key Laboratory of Green Chemical Technology and Processes Engineering School of Chemistry and Chemical Engineering Tiangong University Tianjin 300387 China; ^4^ School of Chemistry and Chemical Engineering Anhui University Hefei, Anhui 230601 China

## Abstract

Highly dispersed bimetallic atomic‐scale catalysts have garnered significant attention in syngas conversion filed due to the synergistic effects of the precisely structured bimetallic site, which facilitate the effective activation of CO. Despite their potential, synthesizing these catalysts to meet the specific application requirements remains challenging. Herein, various bimetallic catalysts were synthesized through the pyrolysis of the bimetallic ZIF precursors which were prepared by *in situ* doping of different metals (Mn, Fe, Co, Ni and Cu) into the ZIF‐8 structure. In the presence of a highly dispersed and highly loaded Zn, the doping content in the ultimate second metallic catalysts varied between 0.15–1.20 wt % for different metals. The catalysts were systematically characterized using XRD, BET, TEM, XPS, Raman, ICP, and H_2_‐TPD techniques. Among them, the Zn−NC regulated with Cu or Ni exhibited superior catalytic performance. Notably, the Cu−Zn−NC catalyst showed the highest activity, achieving a CO conversion of 32.8 % and optimal DME selectivity approaching 95.2 % in CO hydrogenation reactions. These enhanced performance metrics were attributed to the synergetic effects of bimetallic components. The incorporation of Cu not only preserved the original Zn−N structure but also preserved the catalytic performance unchanged. This preparation strategy is expected to filter out new research targets to use in diverse catalytic applications.

## Graphical abstract

To enhance the catalytic performance of Zn−NC in the hydrogenation of CO to dimethyl ether, highly dispersed bimetallic catalysts are synthesized through the pyrolysis different metal‐doped ZIF. The incorporation of secondary metals (Mn, Fe, Co, Ni, Cu) leads to distinct variations in catalytic performance. Among them, the introduction of Cu significantly improves catalytic activity.

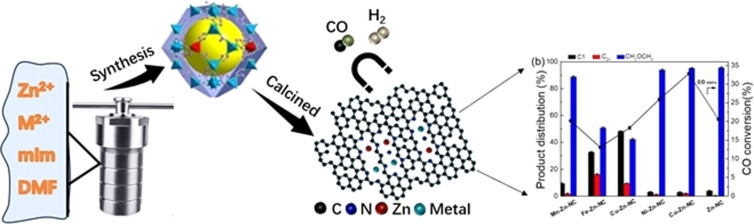

## Introduction

Dimethyl ether (DME) is one of the most useful and valuable chemicals derived from CO hydrogenation, with extensive industrial applications.[^1^, ^2^] Beyond its traditional usages, DME has attracted a great deal of attention as energy carrier, especially being a potential substitute for diesel fuel due to its non‐toxic nature and smokeless combustion properties.[^3^, ^4^] Therefore, DME could play an important role as a chemical intermediate or feedstock in methanol‐DME economy. It is of great practical importance to develop efficient DME catalysts in syngas conversion chemistry.

Typically, DME can be produced by a two‐step process: first, syngas is converted to methanol over metal sites, followed by dehydration to DME over solid acids.[^5^, ^6^] Compared with this indirect synthesis of DME, one‐step methods offer not only thermodynamic advantages, but also lower investment costs, making them more economical. Recently, we reported that the ZIF‐8 derived Zn−NC catalyst at atomic scale has the ability to catalyze the CO hydrogenation to produce DME in a single step, with 20.6 % CO conversion and 95.6 % DME selectivity.^[7]^ The advantage of Zn−NC for catalyzing CO hydrogenation to DME is that it is straightforward and efficient, but the CO activation ability is limited. Although highly dispersed Zn−N catalysts exhibit higher efficacy and promising DME selectivity, the development of new Zn‐based catalysts with superior CO hydrogenation performance remains a great challenge. One of the ways to break the dilemma is to introduce a second metal that can fine tune the active sites.

Highly dispersed bimetallic catalysts leverage adjacent dual metal sites for synergistic and functional complementarity, and can break the limitation of single metal‐N sites by enhancing CO activation, so as to attain better performance.^[8]^ Currently, a variety of diatomic catalysts have been reported in the field of catalysis. For example, Zn−Fe,[^9^, ^10^] Zn−Co,[^11^, ^12^] Zn−Ni,^[13]^ and Zn−Cu[^14^, ^15^] have been reported to show more synergistic effect and better catalytic performance than that of single metal catalysts. Therefore, synthesizing highly dispersed metallic catalysts with dimer sites is a practical strategy to achieve interatomic synergistic effects, offering a promising technique to overcome existing limitations and maximize the catalytic activity for CO hydrogenation. However, due to high surface free energy of atomic‐scale dispersed catalysts, aggregation is more likely to occur.[^16^, ^17^] The preparation of highly dispersed bimetallic catalysts with excellent catalytic performance remains a challenge.

ZIF‐8, which consists of zinc atoms coordinated with imidazolate ligands, offers several advantages, including tunable porosity and chemical versatility. In our previous work, we successfully synthesized ZIF‐8 and utilized it as a precursor to obtain a single‐atom Zn−NC catalyst for DME production. The ionic radii of Mn, Fe, Co, Ni and Cu are similar to that of Zn, theoretically allowing them an equal chance of coordination with nitrogen when incorporated into the ZIF structure synthesis. In fact, numerous bimetallic catalysts have been prepared using this technique.[^18^, ^19^, ^20^] In this work, a series of M−Zn−NC (M=Mn, Fe, Co, Ni or Cu) catalysts were prepared by pyrolyzing the bimetallic ZIF structure materials. For comparison, Zn‐free M−NC (M=Mn, Fe, Co, Ni or Cu) catalysts were also prepared, but they exhibited poor performance in DME formation, suggesting that the introduction of the second metal acts as an accelerant rather than as a main active site. Among M−Zn−NC catalysts, only Ni−Zn−NC and Cu−Zn−NC displayed enhanced catalytic performance, while the other bimetallic catalysts showed no significant improvement.

## Experimental

### Chemicals and Materials

Aqueous solutions of Mn(NO_3_)_2_ (50 %) and 2‐methylimidazole (98 %) were purchased from Aladdin. Fe(NO_3_)_3_ ⋅ 9H_2_O (98 %), Co(NO_3_)_2_ ⋅ 6H_2_O (98.5 %), Ni(NO_3_)_2_ ⋅ 6H_2_O (98 %), Cu(CH_3_OO)_2_ ⋅ H_2_O (99 %) and Zn(NO_3_)_2_ ⋅ 6H_2_O (98.5 %) were provided by Sinopharm Chemical Reagent Co., Ltd, China. N,N‐dimethylformamide (DMF) (99.8 %) and methanol (99.8 %) were supplied by Innochem Co., Ltd, China.

### Catalysts Preparation

#### Preparation of M−Zn−NC (M=Mn, Fe, Co, Ni, Cu)

Various M−Zn−NC catalysts were prepared by calcining bimetallic M−Zn ZIF precursors, which were synthesized using a solvothermal method. A typical procedure for preparing Mn−Zn−NC is described. 1.5 g of Mn(NO_3_)_2_ solution, 4.5 g of Zn(NO_3_)_2_ ⋅ 6H_2_O and an appropriate amount of 2‐methylimidazole were dissolved in 150 ml of DMF under continuous stirring at room temperature for 16 hours to ensure a full dissolution of Zn and Mn precursors in DMF. The mixture was then transferred to a Teflon autoclave and heated at 140 °C for 20 h in a furnace. The resulting bimetallic precursor was washed four times with DMF and methanol, then dried at 65 °C for 20 h, and then calcined for 5 h at 650 °C, and named as Mn−Zn−NC. Other M−Zn−NC (M=Fe, Co, Ni and Cu) were also prepared by the same procedure as Mn−Zn−NC but using different metal salts, i. e., Fe(NO_3_)_3_ ⋅ 9H_2_O, Co(NO_3_)_2_ ⋅ 6H_2_O, Ni(NO_3_)_2_ ⋅ 6H_2_O, and Cu(CH_3_OO)_2_ ⋅ H_2_O respectively. In all cases, an M/Zn molar ratio of 1/10 was used in the synthesis process.

#### Preparation of M−NC (M=Mn, Fe, Co, Ni, Cu)

The boiling point of the metal elements in the M−Zn−NC catalysts are higher than that of 907 °C for Zn, so direct evaporation of Zn was chosen to prepare M−NC catalysts. For example, Mn−NC was synthesized by calcining Mn‐doped ZIF at 1000 °C under a N_2_ atmosphere. Similarly, the collected Fe‐, Co‐, Ni‐, and Cu‐doped ZIF were heated at 1000 °C in a quartz tube under a N_2_ atmosphere for 10 h to produce the corresponding M−NC catalysts, designated as Fe−NC, Co−NC, Ni−NC, and Cu−NC, respectively.

### Catalyst Characterization

X‐ray diffraction (XRD): the crystal structure of samples was obtained using XRD with Co Kα radiation. The diffractometer was carried out at 35 kV and 40 mA. Scans were recorded in 2 theta range of 10° to 100°, with step sizes and each time of 0.02° and 0.2 s, respectively. N_2_ adsorption desorption method: the specific surface area and pore size of information were obtained by Brunauer‐Emmett‐Teller method (BET) using a Micromeritics ASAP 2420 equipment. The analysis involved two steps: (1) Degassing: All samples were degassed under vacuum at 90 °C for 8 h to removed adsorbed impurities. (2) Analysis: Nitrogen was used as adsorption medium, and liquid nitrogen was provided to measure the adsorption and desorption isotherms. The total specific surface area and the total pore volumes were then calculated from the desorption branch of the N_2_ adsorption isotherm using the BET equation and the BJH equation, respectively. Inductively coupled plasma mass spectrometry (ICP‐OES): ICP‐OES was used to measure the actual amounts of metals in the catalysts. For sample preparation, the weighted samples were first calcined in a muffle furnace to remove carbonaceous materials, and the remaining metal was dissolved in HCl, after which the volume was fixed in a 250 ml volumetric flask for testing. A Thermo Icap 6300 (ICP‐OES) (USA) was used. Raman: Raman spectroscopy measurements were performed to get information about carbon structure, phase composition, crystallinity, and molecular interactions in the M−Zn−NC samples. A HORIBA LabRAM HR was used. X‐ray photoelectron (XPS): XPS was used to study the chemical state of elements in the catalysts using a US Thermo Escalab 250 Xi spectrometer with a monochromatic Al Kα as X‐ray radiation source. Transmission electron microscopy (TEM): The elemental composition, morphology and particle size distribution were observed by TEM and EDX analysis. Talos^TM^ 200 A from FEI company was used. Temperature‐programmed desorption of H_2_ (H_2_‐TPD): The adsorption and desorption of H_2_ at the active sites of catalyst was studied by H_2_‐TPD on a Micromeritics AutoChem ASAP 2920. 100 mg of catalyst was loaded into a U‐type quartz tube reactor. The catalyst was heated at 400 °C for 2 h with Ar gas (50 ml/min), and then, the temperature was cooled to −50 °C. The H_2_ flow was maintained for 30 min at −50 °C, followed by flushing with Ar gas for 30 min. The TPD process was carried out by heating the sample from −50 to 800 °C at a constant heating rate under an Ar flow. The signals were detected by a Pfeiffer Omnistar Quadrupole mass spectrometer and the m/z=2 was selected for H_2_‐TPD profiles.

### Catalytic Performance

The catalytic performance of catalysts was carried out in a fixed bed reactor. 1.0 g of catalyst was placed into the isothermal zone of the reactor. The remaining room of reactor was filled with SiC to ensure catalyst fixation. The reaction system was regulated to 3.0 MPa, H_2_/CO=1, GHSV=2000 ml/g_cat_/h, and the temperature was increased to 350 °C at 0.5 °C /min. The tail gases were analyzed online by gas chromatography. The non‐gaseous products were also analyzed offline by gas chromatography. Data collection began after the system reached steady‐state conditions, which typically required about 20 h of operation. The activity evaluation of the catalyst was mainly assessed based on the conversion rate of CO and the selectivity of Cn and DME, calculated using the following equations.

(1) $$\mathrm{Xco}=\frac{Xcoin-Xcoout}{Xcoin}\;\times 100\;\%$$

(2)

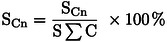



(3)

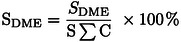




Equation (1): the ratio of converted CO to total number of reactants in the feed gas; Equation (2): the selectivity of CH_4_, C_2_^+^, the weight of the resulting product is the percentage of the total hydrocarbon; Equation (3): the selectivity of CH_3_OCH_3_, the weight of product is the percentage of the total hydrocarbon.

## Results and Discussion

### Structural Information

**Figure 1:**
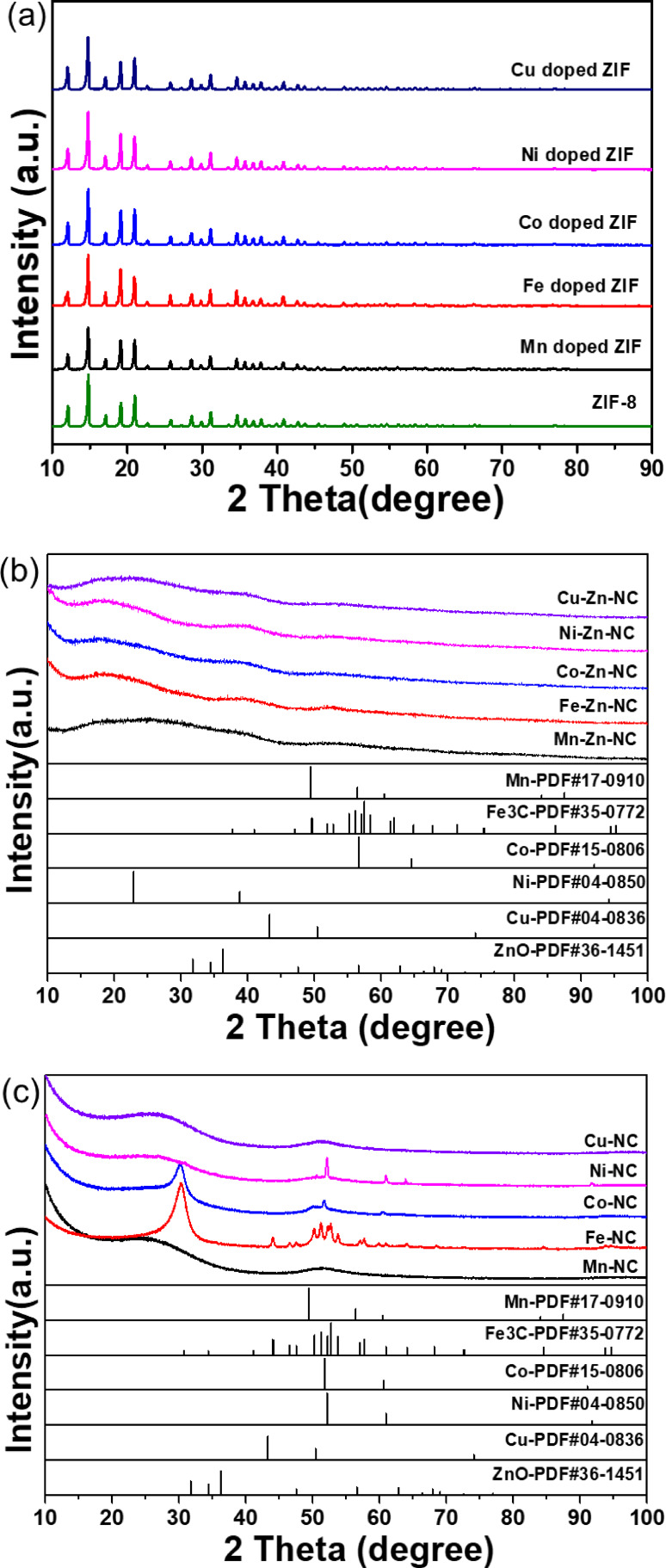
Powder XRD patterns of (a) M‐doped ZIF and ZIF‐8, (b) M−Zn−NC (M=Mn, Fe, Co, Ni and Cu), and (c) M−NC (M=Mn, Fe, Co, Ni and Cu).

The bimetallic M−Zn−NC catalyst was fabricated using metal‐doped ZIF as a precursor. The unique porous structure of the ZIF framework not only provides enough space to accommodate additional metal atoms but also limits the aggregation of atoms because of chemical bonding.[^21^, ^22^] The XRD patterns of the metal‐doped ZIF precursors (Figure 1a) and FT‐IR spectra (Figure S1) demonstrate that the structural integrity of the ZIF‐8 framework was preserved after the introduction of the second metal within the catalyst structure. No additional phases or species were observed, confirming the high dispersion of the second metal within the catalyst structure. Subsequently, the M‐doped ZIF precursors were pyrolyzed at 650 °C under a N_2_ atmosphere to obtain the M−Zn−NC catalysts. The elemental composition and textural properties of these catalysts are summarized in Table 1. Although the same molar ratio of M/Zn was used in the preparation of the catalysts, the amount of the second metal left in the skeleton was different, indicating differences in the hosting capacity of ZIF framework for different metal elements. The actual metal content ranged from 0.15 to 1.20 %, while the N and C contents remained relatively consistent across the catalysts.

**Table 1:** Textural properties of the catalysts.

Sample	Zn/wt %	Mn/wt %	Fe/wt %	Co/wt %	Ni/wt %	Cu/wt %	C %m/m	N %m/m	S_BET_ (m^2^/g)	S_micro_ (m^2^/g)	V_pore_ (cm^3^/g)	D_pore_ (nm)
Mn−Zn−NC	28.20	1.09					38.3	24.2	344	322	0.01	65.2
Fe−Zn−NC	31.60		1.20				42.1	19.6	40	21	0.04	26.2
Co−Zn−NC	29.00			0.56			38.9	22.1	390	342	0.02	4.7
Ni−Zn−NC	30.80				0.36		39.1	22.4	533	473	0.02	3.3
Cu−Zn−NC	31.20					0.15	40.1	24.3	432	368	0.01	2.7
Zn−NC	29.20						41.5	24.3	625	595	0.05	3.9
Mn−NC		2.00					79.4	3.1	778	636	0.04	3.3
Fe−NC			2.55				85.4	2.0	345	138	0.22	3.9
Co−NC				1.84			85.1	3.9	605	361	0.18	3.8
Ni−NC					1.20		78.2	6.7	806	685	0.02	38.5
Cu−NC						1.99	87.6	4.9	720	560	0.05	3.7

The BET surface area and pore size distribution are crucial factors influencing the exposure of active sites. According to the IUPAC classification, all M−Zn−NC catalysts exhibit a similar H4‐type hysteresis loop, consistent with Zn−NC catalyst (Figure S2 and S3). However, the BET surface areas of these catalysts vary significantly, ranging from 40 to 533 m^2^/g (Table 1). Thereinto, Fe−Zn−NC exhibits a relatively smaller BET surface area, which can be attributed to the surface deposition of Fe species on the network skeleton. This conclusion is supported by the absence of Fe metal or related compound phase in both corresponding XRD and TEM characterization. These analytical results show that the observed reduction in specific surface area is not associated with the formation of metal particles. the Ni−Zn−NC and Cu−Zn−NC catalysts exhibited the highest BET surface area of over 430 m^2^/g, with an average pore size close to 3.0 nm, which can expose more active sites and provide more transport channels for CO and DME product. These enhanced properties may be attributed to the formation of localized interactions between the Ni/Cu and Zn sites, which contribute to the structural and textural differences observed in these catalysts.

XRD analysis provides valuable insights into the structure properties, phase, preferred crystal orientations, average grain size, crystallinity, tension, and crystal defects of the catalysts (Figure 1). Figure 1b displays the XRD patterns of the M−Zn−NC catalysts. There are no significant diffraction peaks in each catalyst, which may be attributed to the small particle size or the high dispersion of metal compounds in the pyrolysed samples. The M−NC catalysts were synthesized by evaporating Zn from M‐doped ZIF precursors. After removing Zn from the M‐doped ZIF precursors, the Mn−NC and Cu−NC still showed highly dispersive states, while for Fe−NC, Co−NC, and Ni−NC, crystallized Fe_3_C, Co, and Ni phases were formed (Figure 1c). Given the properties of Fe, Co, and Ni, these metal atoms are more likely to aggregate to form metal nanoparticles. The Zn−N bond is primarily formed in the ZIF framework, which is the key coordination that overcomes the aggregation of other atoms after pyrolysis.^[23]^

**Figure 2:**
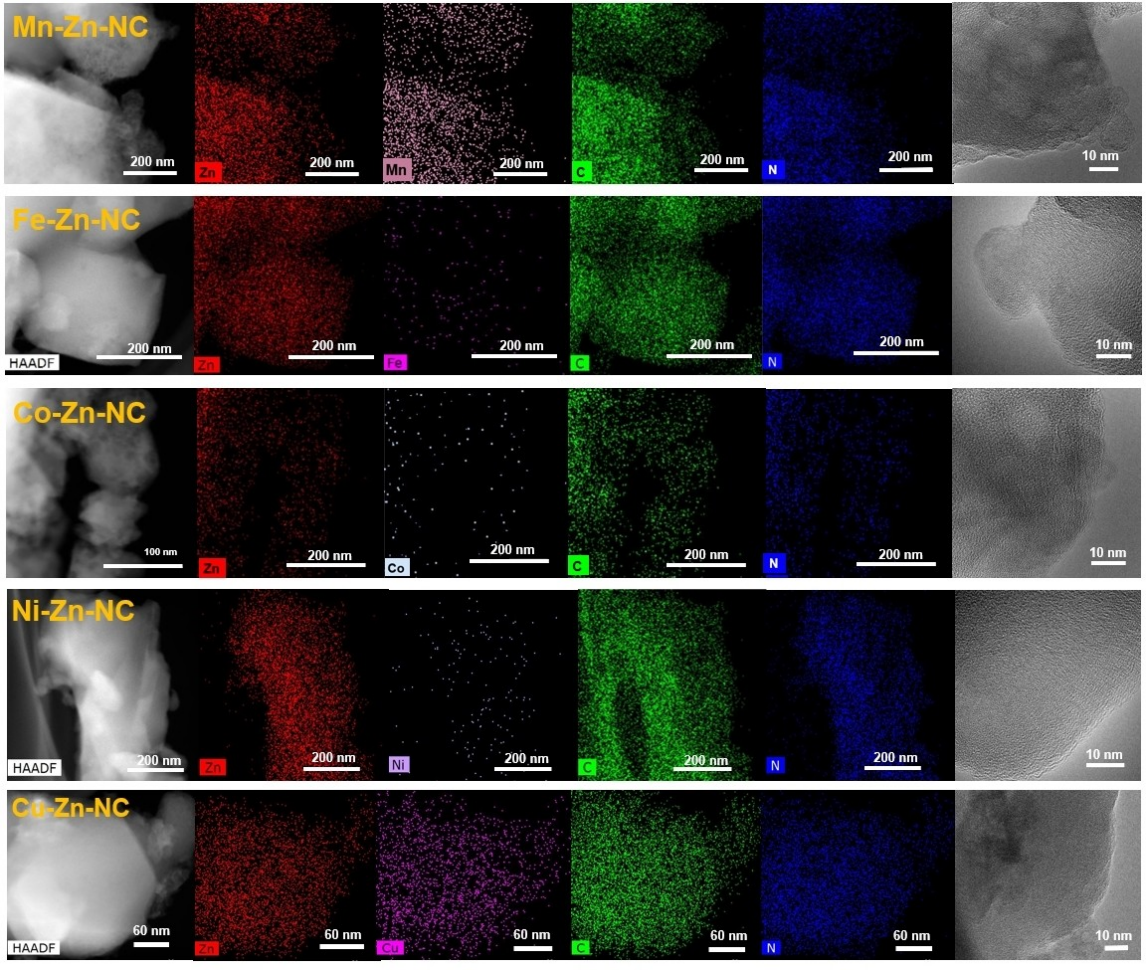
TEM image of prepared catalysts and corresponding EDX elemental mapping images for Mn−Zn−NC, Fe−Zn−NC, Co−Zn−NC, Ni−Zn−NC and Cu−Zn−NC catalysts.

EDX elemental mapping reveals that Zn, C, N and the other metal species are uniformly dispersed within the carbon supports (Figure 2). Transmission electron microscopy (TEM) profiles exhibit that there are no significant metal nanoparticles or alloy phases in all M−Zn−NC catalysts, while also indicating the presence of amorphous carbon in all samples. Notably, despite the relatively higher content of Mn and Fe, no distinct lattice fringes appeared in the high‐resolution TEM images. Therefore, these findings indicate that the adopted preparation method successfully yields bimetallic catalysts with highly homogeneous dispersion of metal species, highlighting the effectiveness of the synthesis approach in preventing metal aggregation.

**Figure 3:**
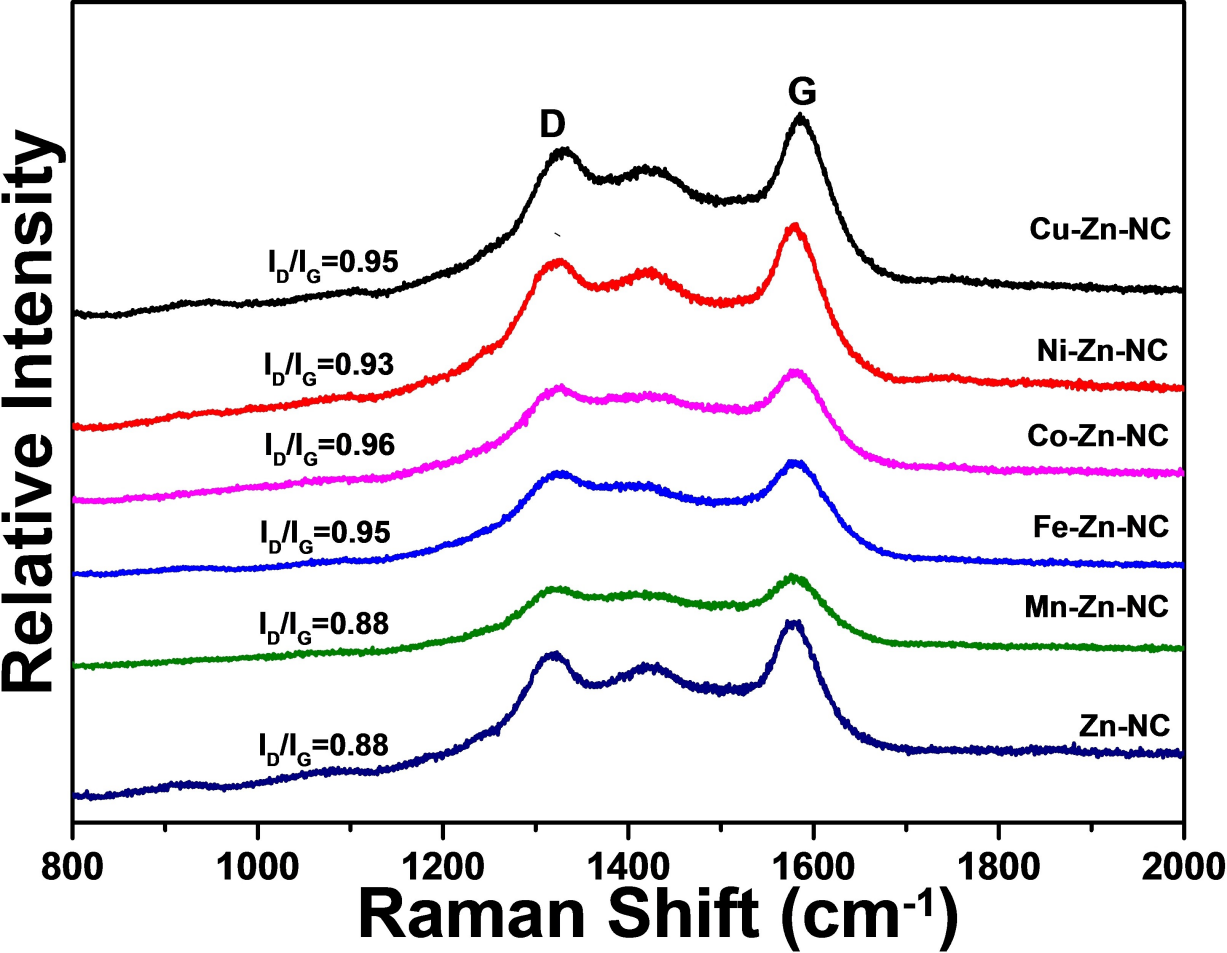
Raman spectra of Mn−Zn−NC, Fe−Zn−NC, Co−Zn−NC, Ni−Zn−NC, Cu−Zn−NC, Zn−NC.

According to the Raman spectra (Figure 3), the obvious peaks corresponding to disordered carbon (D band) and graphitic carbon (G band) peaks were located at 1350 cm^−1^ and 1580 cm^−1^, respectively. The relative content of amorphous carbon to graphite carbon can be quantified using the intensity ratio of I_D_/I_G_, as referenced in previous study.^[24]^ Compared to the I_D_/I_G_ intensity ratio of 0.88 of Zn−NC, the I_D_/I_G_ intensity ratio for M−Zn−NC increased (0.95 of Fe−Zn−NC, 0.96 of Co−Zn−NC, 0.93 of Ni−Zn−NC, 0.95 of Cu−Zn−NC), indicating that the metal doping enhances carbon disorder in the M−Zn−NC catalysts. These defect sites are likely to provide additional active sites, which may contribute to the improved catalytic activity. In addition, similar to the Zn−NC catalyst, the Ni−Zn−NC and Cu−Zn−NC catalysts exhibited a characteristic peak at 1420 cm^−1^, attributed to heterocyclic carbons structure.[^25^, ^26^] This suggests that the bimetallic active sites were uniformly deposited with the N‐doped carbon support, further supporting the structure homogeneity of the catalysts.

### Catalytic Performances

**Figure 4:**
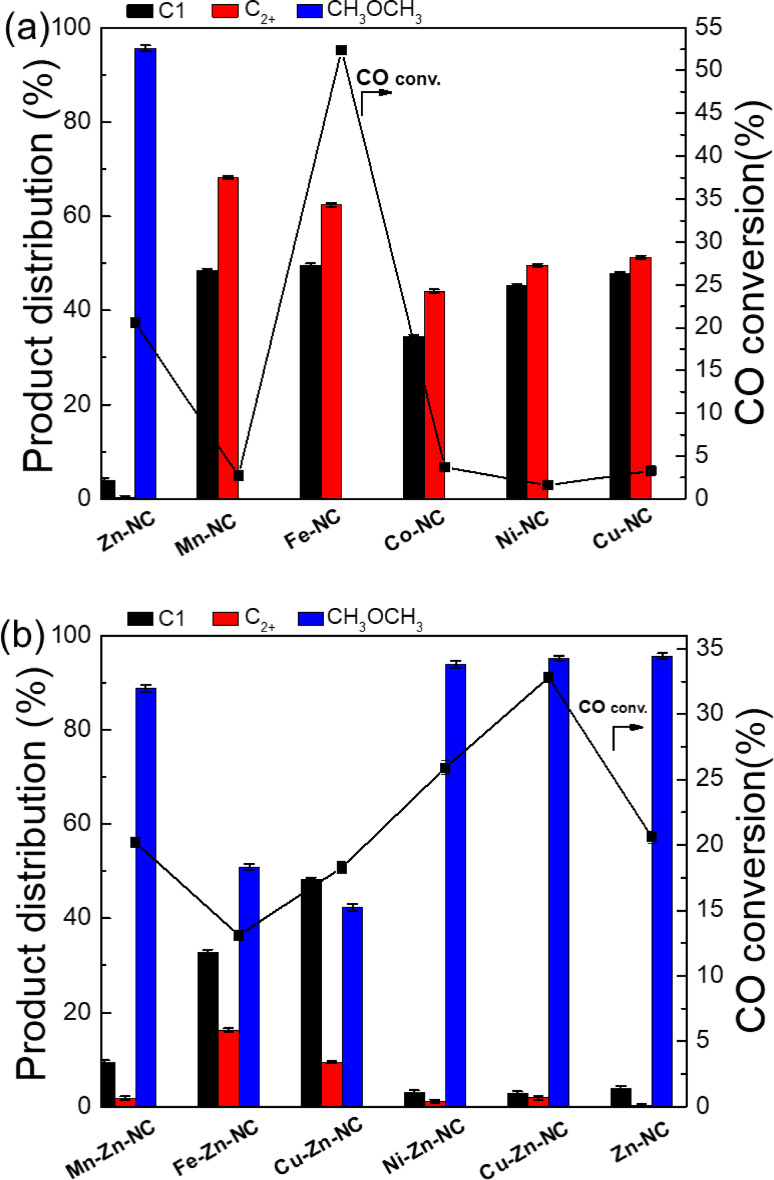
One‐step conversion of CO to DME reaction performance of a) M−Zn−NC and b) M−NC catalysts. (Reaction conditions: catalyst (1.0 g), reaction temperature (350 °C), reaction pressure (3 MPa), H_2_/CO=1; Conv=conversation, Sel=selectively. The corresponding error bars represent the standard deviation calculated from the five consecutive data points collected after the system reached a stabilized state.)

Catalytic tests were carried out in a fixed‐bed reactor to evaluate the CO hydrogenation performance of the M−Zn−NC catalysts, with the results presented in Figure 4. For comparison, single‐metal catalysts, including Zn−NC, Mn−NC, Fe−NC, Co−NC, Ni−NC and Cu−NC, were also evaluated (Figure 4a). Among them, the catalytic tail gas analysis of Zn−NC demonstrated that CH_3_OCH_3_ was exclusively detected on the HP‐PONA column, while both low‐carbon hydrocarbons and CH_3_OCH_3_ were identified on the GasPro column, confirming CH_3_OCH_3_ as the primary reaction product (Figure S4). The Zn−NC catalyst exhibited significant catalytic performance, achieving a CO conversion of 20.6 % and DME selectivity of 95.6 %. These results indicate that the Zn species as the primary active site for CO conversion to DME. In contrast, all other M−NC catalysts, except Fe−NC, showed CO conversion below 5 % and no DME was generated in the products. Although the CO conversion of the Fe−NC catalyst was 52.4 %, the products were predominantly methane and C_2+_ hydrocarbons. This indicates that Fe_3_C acts as an active site for CO hydrogenation to alkanes instead of DME.

**Figure 5:**
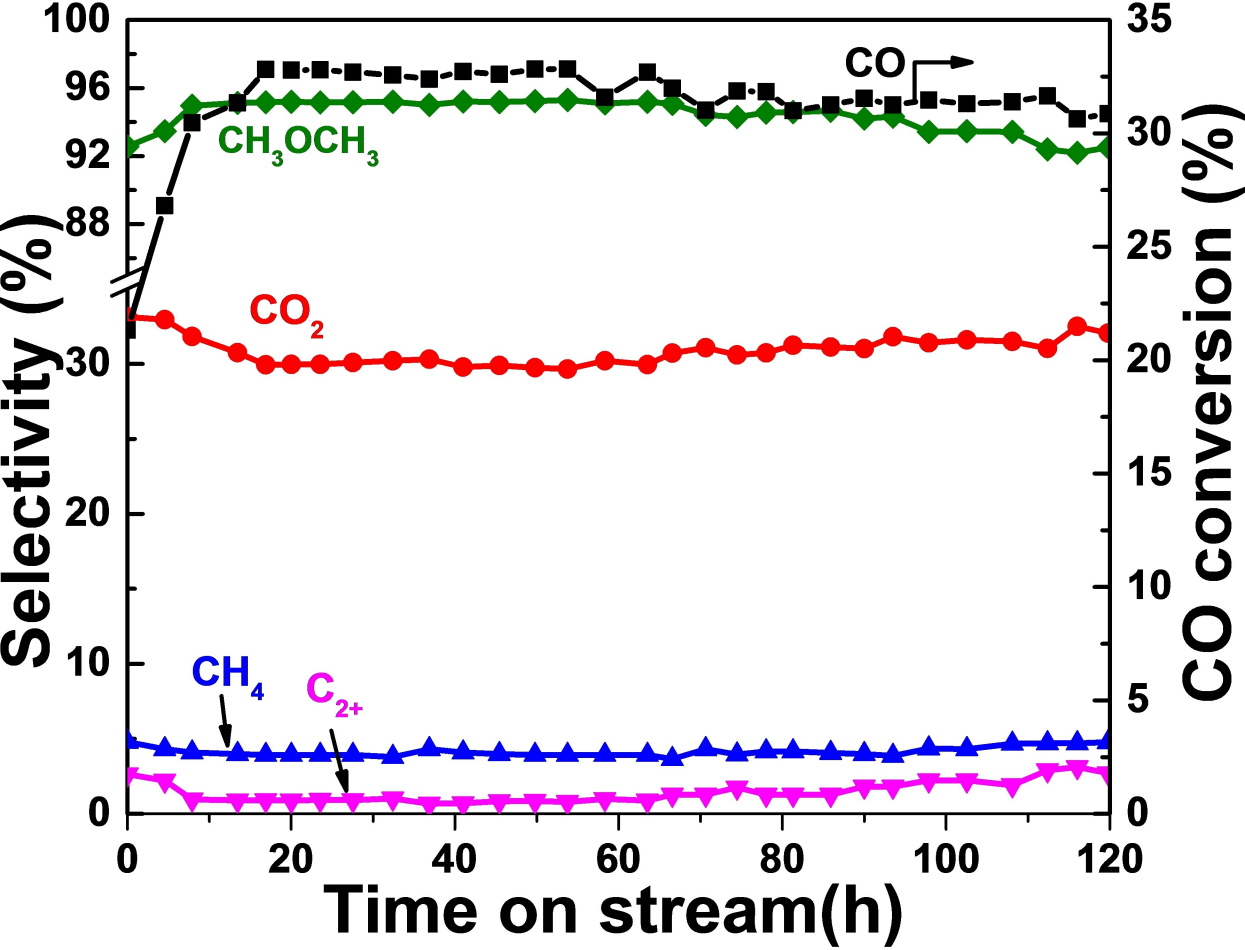
Stability of the Cu−Zn−NC catalyst in the CO hydrogenation to DME reaction. Conditions: 1.0 g of the catalyst, 350 °C, 3 MPa, H_2_/CO=1.

For the bimetallic M−Zn−NC catalysts (Figure 4b), Mn−Zn−NC, Fe−Zn−NC, and Co−Zn−NC exhibited CO conversion of 20.2 %, 13.1 %, and 18.3 %, respectively. However, their DME selectively were below 90 %, with increased methane formation, demonstrating that the introduction of Mn, Fe, and Co has no positive effect on DME production. In contrast, the catalytic performance of Ni−Zn−NC and Cu−Zn−NC was better than that of Zn−NC, achieving CO conversions of 25.9 % and 32.8 %, respectively, both higher than that of Zn−NC (20.6 %). These results confirm that the introduction of Ni or Cu as a second metal preserves the high specific surface area, ensuring unobstructed access to active sites. These bimetallic structures synergistically promote the reactivity of the Zn−N active sites, leading to enhanced catalytic performance. Among them, Figure 5 exhibits the catalytic stability of the Cu−Zn−NC catalyst in 120 h reaction. The introduction of Cu enhances the CO conversion to 32 %, and this performance can be maintained up to 55 h. As the reaction time increases, a slightly decline in catalytic activity was observed. However, the CO conversion and DME selectivity can be maintained at approximately 30 % and 92 %, respectively. In brief, the incorporation of metal dopants into the catalyst significantly modulates the active sites,[^27^, ^28^] leading to a positive impact on the overall catalytic performance.

### The Surface Structure Properties of Catalysts

**Figure 6:**
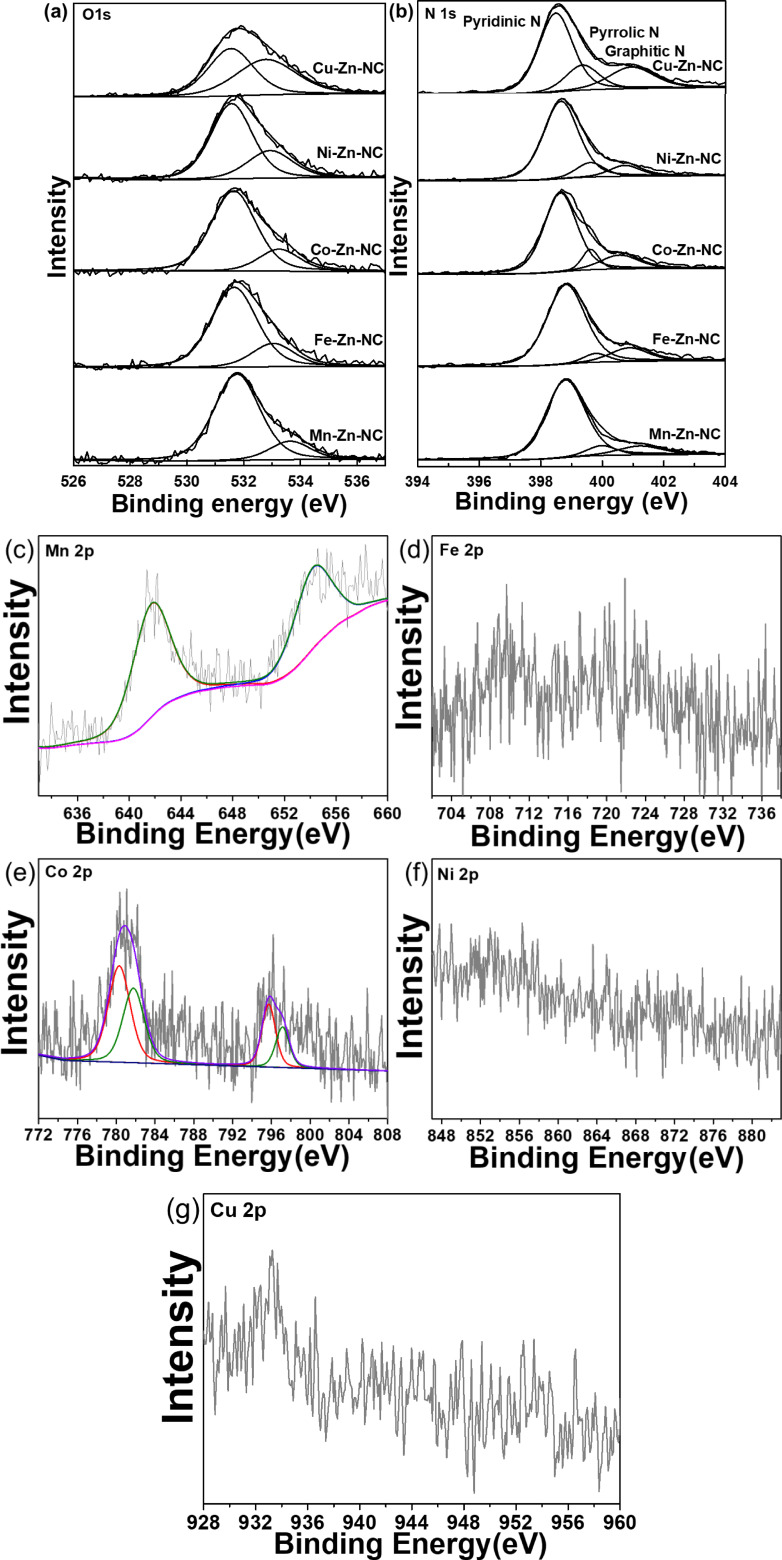
(a) High‐resolution XPS spectra of O1s of M−Zn−NC. (b) High‐resolution XPS N1s spectra of M−Zn−NC. (c) High‐resolution XPS Mn 2p spectrum of Mn−Zn−NC. (d) High‐resolution XPS Fe 2p spectrum of Fe−Zn−NC. (e) High‐resolution XPS Co 2p spectrum of Co−Zn−NC. (f) High‐resolution XPS Ni 2p spectrum of Ni−Zn−NC. (g) High‐resolution XPS Cu 2p spectrum of Cu−Zn−NC.

X‐ray photoelectron spectroscopy (XPS) analysis was conducted to study the specific chemical composition and elemental states of M−Zn−NC catalysts. In the high‐resolution O 1s spectra (Figure 6a), M−Zn−NC catalysts showed two distinct peaks at binding energies of 531.4–531.6 eV and 533–534 eV, corresponding to adsorbed oxygen and surface O species, respectively. Notably, no metal‐oxygen peak was observed at 530.0 eV,^[29]^ confirming the absence of metal oxide in the catalysts. In the high‐resolution N 1s spectra (Figure 6b), the N content was found to be similar across all catalysts. Three kinds of N species were identified as pyridinic N, pyrrolic N and graphitic N.[^30^, ^31^] These results demonstrated that metal atoms have an equal chance of anchoring onto the N‐doped carbon framework. In addition, the XPS spectra of M 2p in the M−Zn−NC catalysts (Figure 6c–6 g) exhibited that the second metal atoms exist in cationic states. Therefore, without interference from the metal oxide state, the metal atoms are most likely to coordinate with N in the catalyst.

The solvothermal preparation method can guarantee the existence of Zn within the ZIF‐8 frameworks and allows for the partial introduction of a second metal without affecting the lattice structure of ZIF‐8. Under the condition that the loading second metal is sufficiently low, the metal ions can be uniformly dispersed after pyrolysis of the precursor M‐doped ZIF.

**Figure 7:**
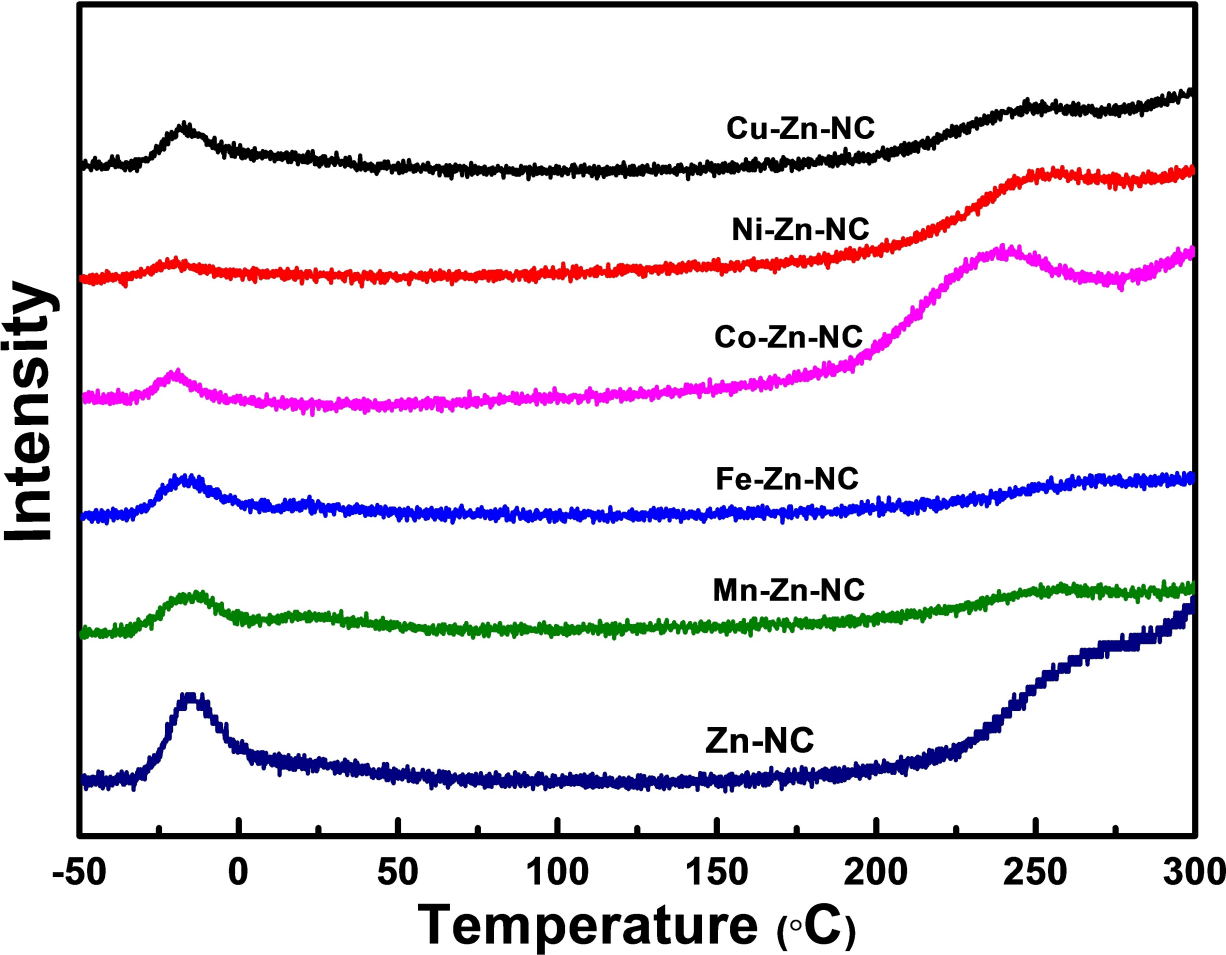
H_2_‐TPD profiles of M−Zn−NC catalysts.

In the H_2_‐TPD profiles (Figure 7), the H_2_ desorption area at −18 °C obviously decreased for M−Zn−NC catalysts compared to the Zn−NC catalyst, manifesting that the bimetallic catalysts M−Zn−NC have a lower ability for adsorption. In other words, the introduction of the second metal tends to make H more readily available on the surface of the catalyst, enhancing its combination with the activated CO. Consequently, the enhanced activity of CO activation leads to higher selectivity for DME production.

### The Effect of Different Metals

The key reaction step in CO hydrogenation is the CO activation to produce DME directly. According to the reported literature,[^32^, ^33^, ^34^] CuZnAl catalysts typically exhibit lower specific surface area (usually no more than 200 m^2^/g), necessitating modifications or increasing components to enhanced the dispersion of active sites and improve the catalytic performance. For examples, as shown in Table S1, CuZnAl catalysts have been modified by the incorporation of metals to increase specific surface areas or by acid‐active compounds to improve the catalytic performance. While these traditional catalysts typically have considerable CO conversion rates, but the product selectivity distribution is complicated by the influence of multiple components, resulting in low DME selectivity. However, the Zn−NC catalyst possesses a significantly larger specific surface area compared to CuZnAl catalysts. According to X‐ray Absorption Fine Structure analysis and theoretical calculations reported in the literature,^[7]^ Zn‐N_3_ sites have been identified as the dominant active site for CO activation. After introduction of Cu, the Cu−Zn−NC catalyst demonstrates enhanced CO activation capability while maintaining product selectivity, as evidenced by the absence of new byproducts under the reaction conditions. Furthermore, the EXAFS analysis of Cu−Zn−NC not only demonstrates the absence of two distinct coordination characteristic peaks characteristic of CuO or Cu_2_O, but also excludes the absence of Cu−Cu coordination at 2.2 Å, aligning with the TEM observations. These findings indicate that Cu−Zn−NC sample does not contain large particles of oxide copper or metallic copper but rather features highly dispersed copper species (Figure S5). Additionally, the Cu−Zn−NC reveals a peak at 2.4 Å, which may correspond to coordination with outer atoms, such as Zn. This enhanced catalytic performance strongly indicates the potential formation of a novel Cu−Zn structural configuration, which likely serve as an optimized active site and thereby significantly promoting CO conversion efficiency. According to the performance of these M−Zn−NC catalysts, increasing appropriate CO hydrogenation metal catalyst can promote the performance. Achieving excellent CO hydrogenation performance needs a synergistic effect of CO active sites. Therefore, the introducing of a second metal enhances the intrinsic activity of Zn−N, creating additional CO active sites and thus improve catalytic performance.

**Figure 8:**
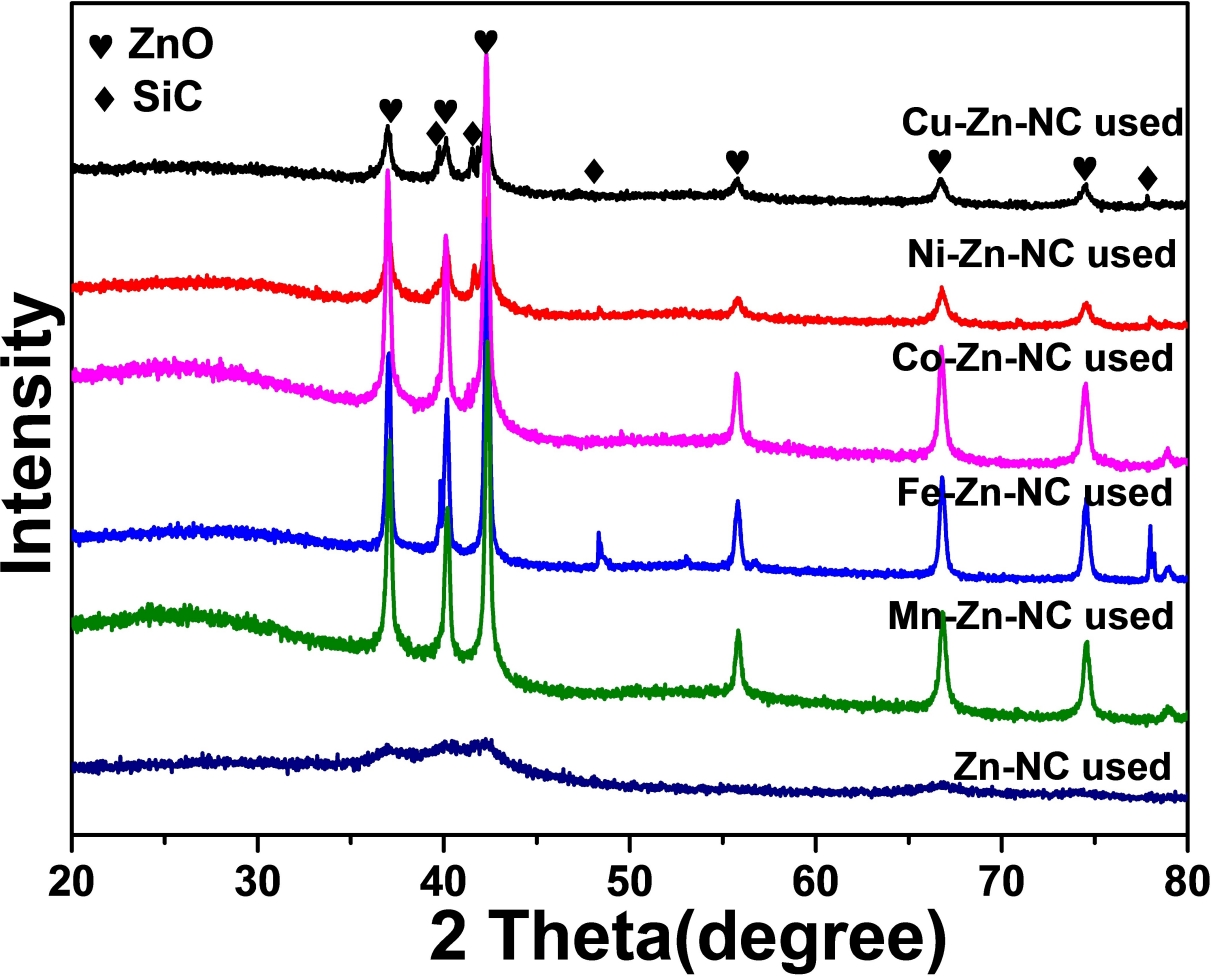
the XRD patterns of M−Zn−NC catalysts after reaction.

**Figure 9:**
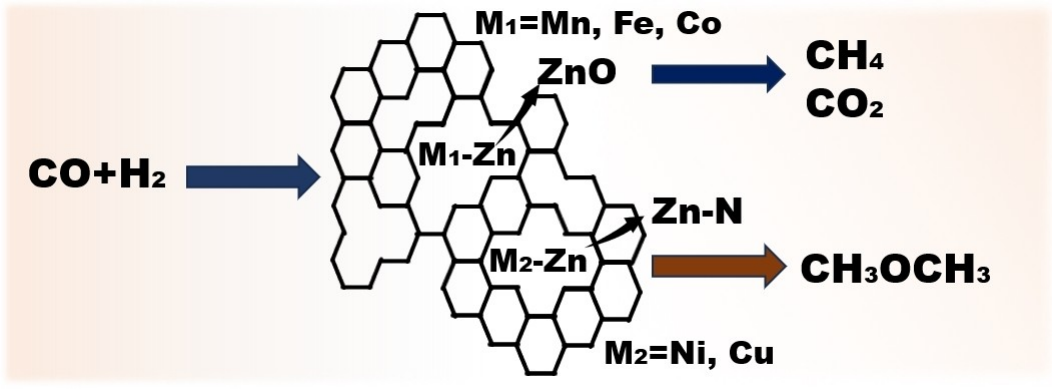
The reaction scheme of the second metal introduced into Zn−NC catalyst.

As the metals are introduced into the Zn−N structure, they tend to coordinate to different states. In our investigation of the phase state of used M−Zn−NC catalysts (Figure 8), the introduction of Mn, Fe, and Co preferentially promoted the formation of ZnO, reducing the Zn−N active sites significantly. But Ni and Cu were less prone to form ZnO, thereby maintaining the original state of Zn−N structure. This phenomenon shows that the introduction of different metals will produce different degrees of state change in the reaction. Consequently, the reaction mechanisms of CO hydrogenation to DME on Zn−N and other M−Zn will be different. The catalytic selectivity of Cu−Zn−NC is similar to that of Zn−NC, indicating that CO can be appropriately activated on Cu−Zn sites under the same reaction conditions. Therefore, the Mn‐, Fe‐, Co−Zn−NC catalysts tend to aggregate to form oxides under high‐temperature reactions, thus reducing the original active sites, which is not conducive for CO conversion to DME and results in poor catalytic performance. The reaction structural changes induced by the introduction of these metals are illustrated in Figure 9.

## Conclusions

In summary, a series of bimetallic catalysts with a highly dispersed state were fabricated for CO hydrogenation to DME reaction. After introducing Mn, Fe, Co, Ni, and Cu to ZIF‐8 framework, the metal ions were more likely to exist in the form of the corresponding elements Zn or N coordination. The addition of Ni and Cu species to Zn−NC not only enhanced CO conversion but also maintained high DME selectivity. Among these catalysts, Cu−Zn−NC and Ni−Zn−NC catalysts exhibited enhanced catalytic performance than that of Zn−NC catalyst. For the Cu−Zn−NC catalyst, the enhancement was due to the presence of a Cu−Zn site with an appropriate coordination structure, which facilitated CO activation and subsequent DME production. Under reaction conditions of 3 MPa, 350 °C and GHSV =2000 ml/g/h, X_**co**_ value increased to 32.8 % and S_DME_ value remained at 95.2 %. The synergistic effect between the Cu−Zn bimetallic sites not only boosted the CO conversion by more than 10 % but also maintained the DME selectivity. Therefore, this work provides a new insight into the design of bimetallic catalysts for syngas conversion and highlights the significant potential of Zn‐based catalysts in this field. Furthermore, it underscores the importance of further research into the development and optimization of such catalytic systems.

## Author Contributions

C.Q. Z. performed the main experiments and wrote the paper draft. Q. C. conducted the data analysis and revised the manuscript. F. Y. and G.W. N. carried out the XRD and H_2_‐TPD tests and provided the analysis. A.T. K. and B.B. M. supervised the project and revised the manuscript. D. L. and C.H. Z. conceived and supervised the project. All authors participated in the discussion of this work.

## Conflict of Interests

There are no conflicts to declare.

1

## Supporting information

As a service to our authors and readers, this journal provides supporting information supplied by the authors. Such materials are peer reviewed and may be re‐organized for online delivery, but are not copy‐edited or typeset. Technical support issues arising from supporting information (other than missing files) should be addressed to the authors.

Supporting Information

## Data Availability

The data that support the findings of this study are available from the corresponding author upon reasonable request.
