# Supplementary material for: Catalytic Performance of Highly Dispersed Bimetallic Catalysts for CO Hydrogenation to DME
Source: Chempluschem. 2025 Mar 12;90(5):e202500010. doi: 10.1002/cplu.202500010 (PMC12105462; doi:10.1002/cplu.202500010)
Supplement: Supplementary file 1 — Supporting Information [file CPLU-90-e202500010-s001.pdf]

ChemPlusChem

Supporting Information

Catalytic Performance of Highly Dispersed Bimetallic Catalysts for CO Hydrogenation to DME

Chunqiu Zhao, Qiang Chang,* Fu Yin, Guowei Niu, Chenghua Zhang,* Dan Liu, Bhekie B. Mamba, and Alex T. Kuvarega*

Supporting information

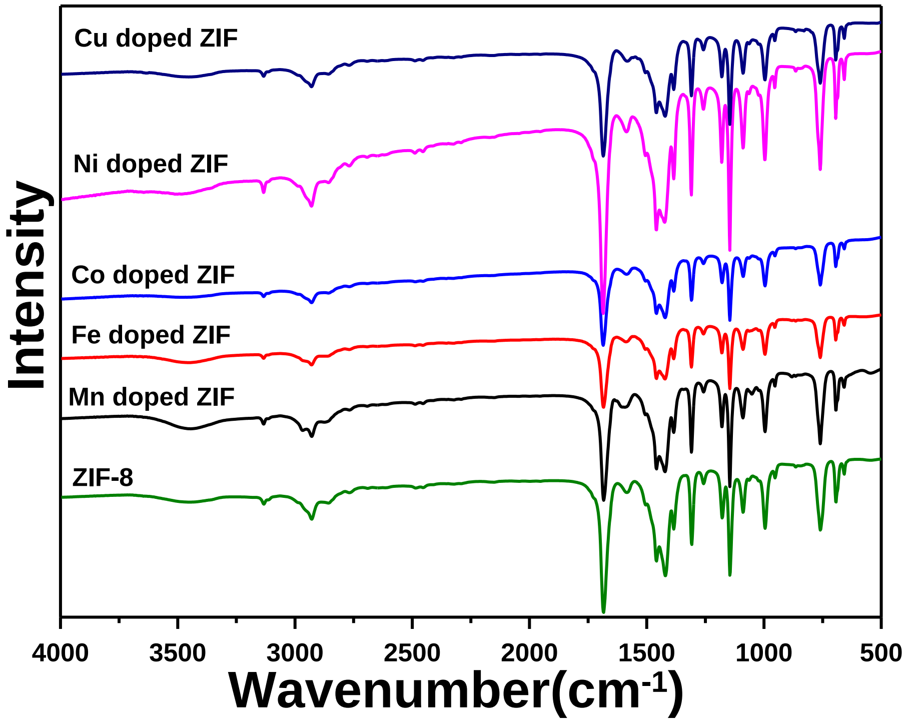

Figure S1. FT-IR spectroscopy over the different metal-doped ZIF precursors.

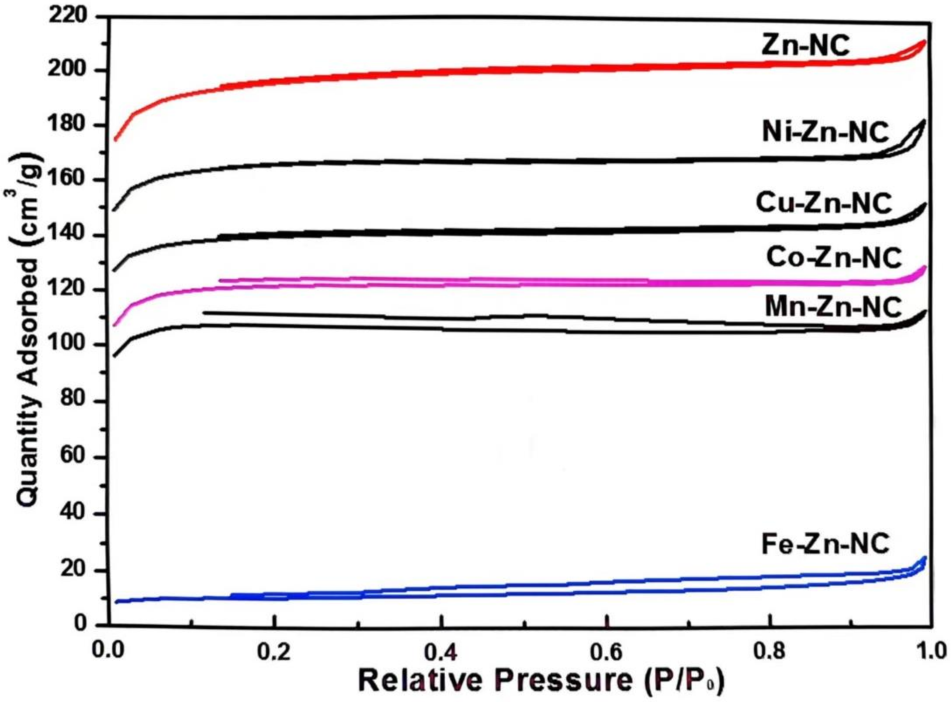

Figure S2. N_2 sorption isotherms for M-Zn-NC catalysts at 77 K.

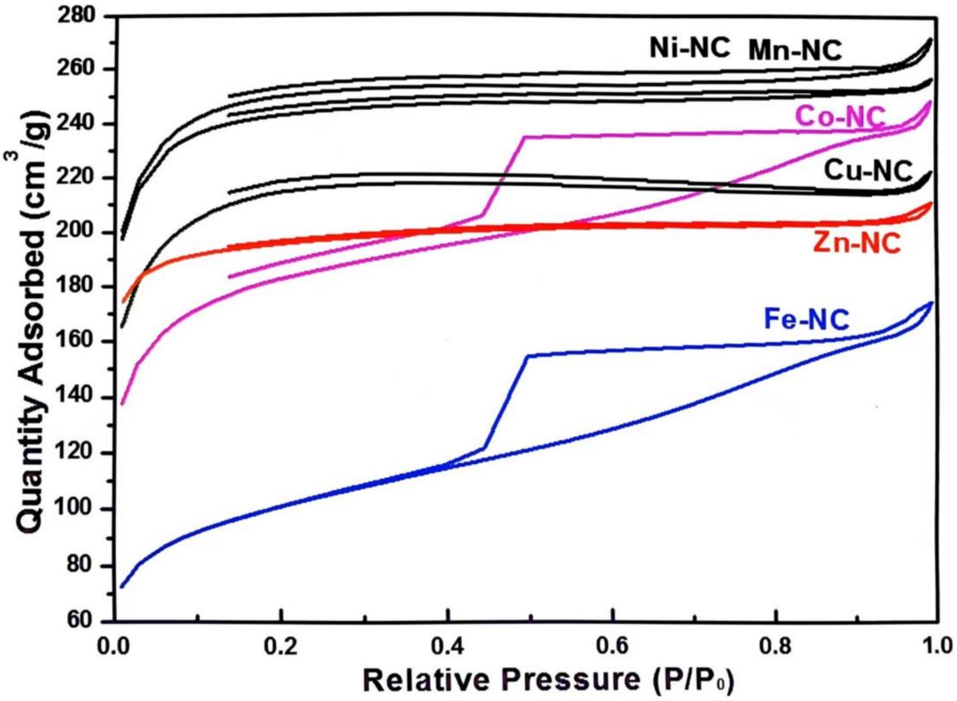

Figure S3. N_2 sorption isotherms for M-NC catalysts at 77 K.

(a)

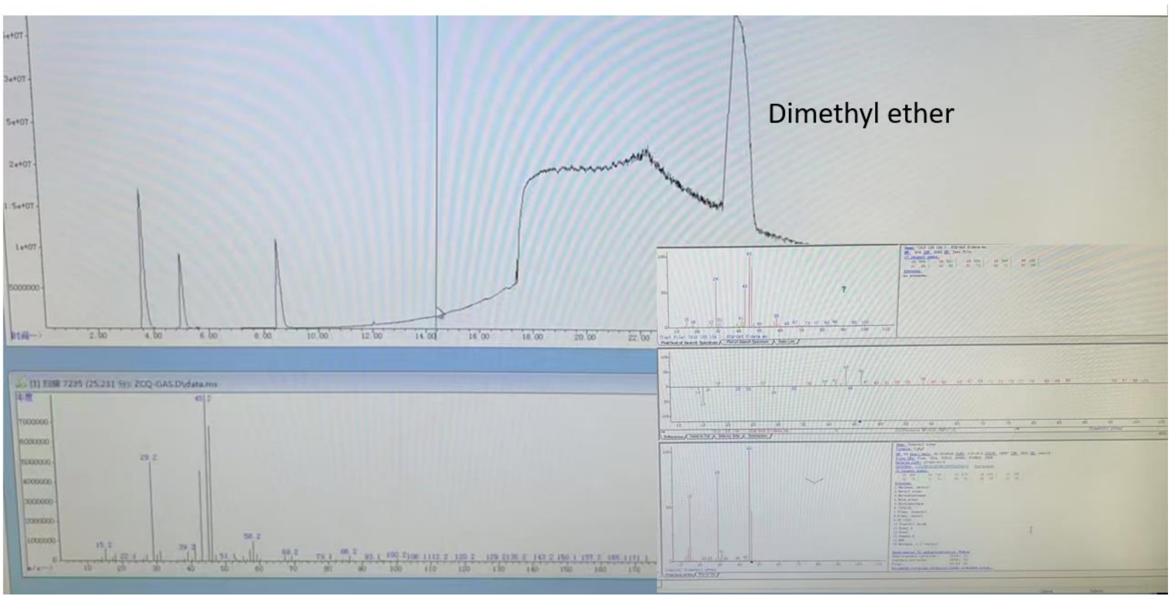

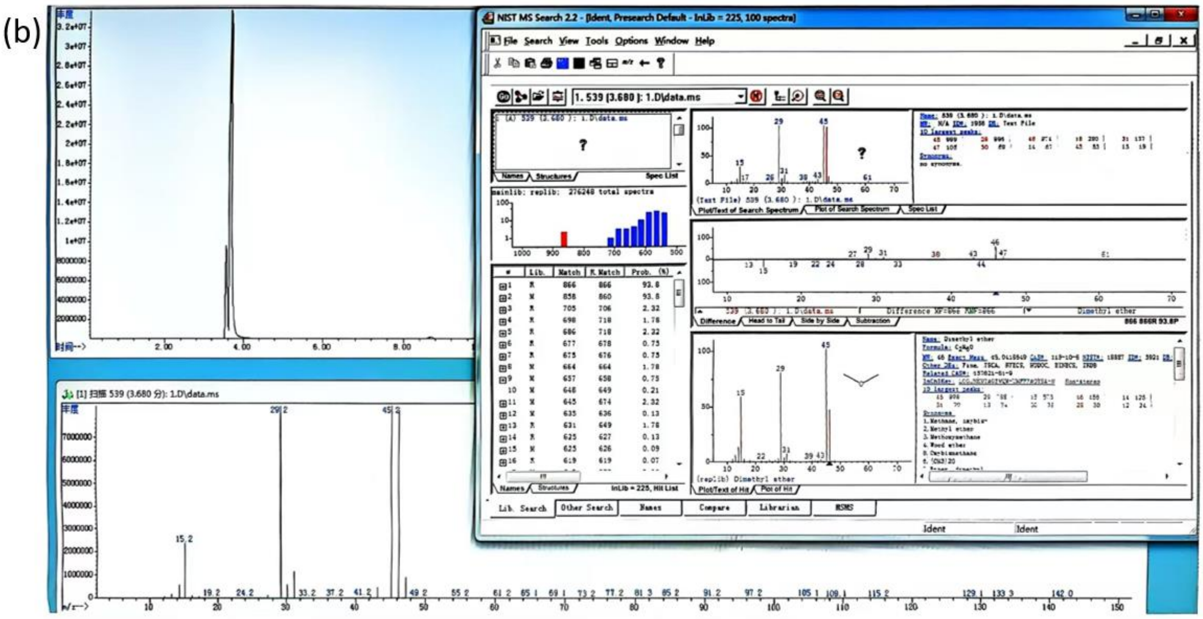

Figure S4. The GC-MS results of product for Zn-NC catalyst, a) the column type is GasPro for the analysis of C_{1-4}, containing dimethyl ether. b) the column type is HP-PONA for the analysis of C_4^+.

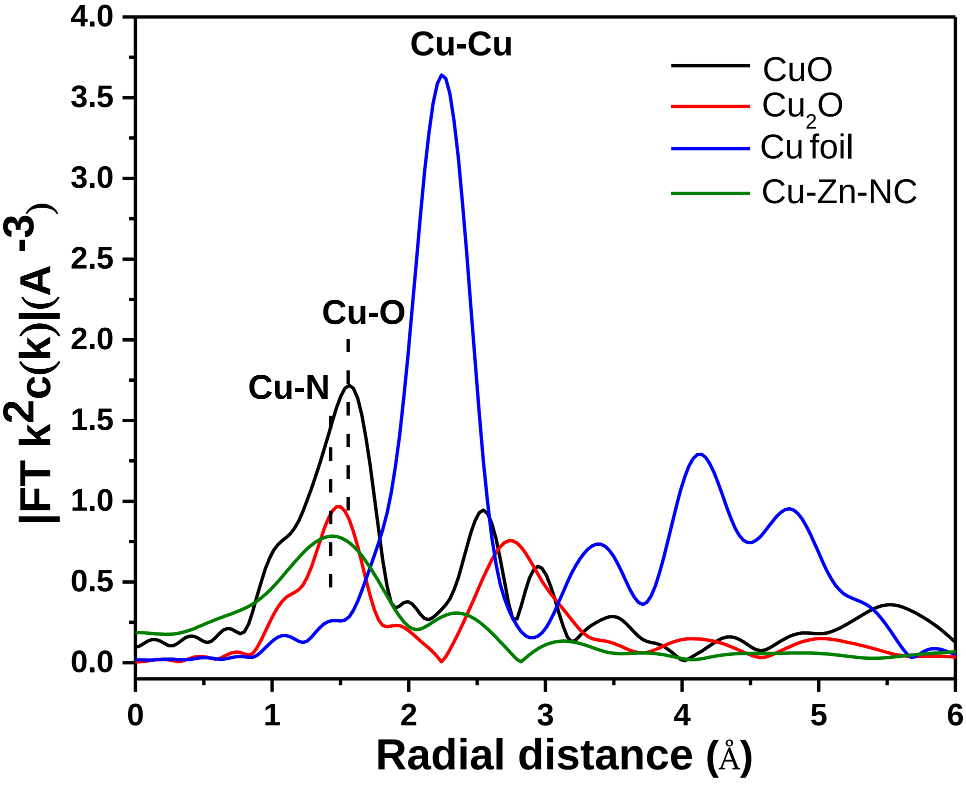

Figure S5. Cu K-edge Fourier transform EXAFS spectra of CuO，Cu_2O, Cu foil and Cu-Zn-NC samples.

Table S1. The CO conversion and DME selectivity performance of the reported literatures on traditional catalysts.

Catalysts	CO conversion (%)	DME selectivity (%)	H_2/CO	T/^{o}C	P/MPa	References
CuZnAl-HZSM-5	57.4	64.0	1	250	3.0	[1]
FCuZnZr25(N)-10Z	39.8	60	1	300	4	[2]
Zr/CuZnAl	79.3	85.8	2	270	5	[3]
Mg-CuZnAl-HZSM-5	96.3	64.5	2	260	4	[4]
CuZnZr/HZSM-5	72.8	83.1	2.2	250	3	[5]
CuZnAl/HZSM-5	48.5	97	1	260	4	[6]
Zn-NC	20.6	95.6	1	350	3	[7]
Cu-Zn-NC	32.8	95.2	1	350	3	This work
Ni-Zn-NC	25.9	93.9	1	350	3	This work

Reference:

[1] X. Guo, F. Liu, Y. Hua, H. Xue, J. Yu, D. Mao, G. L. Rempel and F. T. T. Ng, *Catalysis Today*, 2023, 407, 125-134.

[2] J. Palomo, M. Á. Rodríguez-Cano, J. Rodríguez-Mirasol, T. Corde, Applied Catalysis B: Environmental, 2020

[3] F. Song, Y. Tan, H. Xie, Q. Zhang, Y. Han, Fuel Processing Technology 126 (2014) 88–94.

[4] D. Mao, W. Yang, J. Xia, B. Zhang, Q. Song, Q. Chen, Journal ofCatalysis 230 (2005) 140–149.

[5] K. Sun, W. Lu, F. Qiu, S. Liu, X. Xu, Applied Catalysis A: General 252 (2003) 243–249.

[6] W. Lu, L. Teng, W. Xiao, Chemical Engineering Science 59 (2004) 5455–5464.

[7] C. Zhao, J. Liu, Y. Wei, T. Shi, J. Xue, Q. Chang, S. Sun, D. Liu, A. T. Kuvarega, B. B. Manba, C. Zhang, ACS Appiled Nano Materials, 2024, 7, 2, 2224–2231.
